# Automated CT‐Based Muscle Density Predicts Mortality Regardless of Muscle Area

**DOI:** 10.1002/jcsm.70268

**Published:** 2026-04-02

**Authors:** Adam J. Kuchnia, Glen M. Blake, Matthew H. Lee, Jevin Lortie, Rachel Fenske, John W. Garrett, Perry J. Pickhardt

**Affiliations:** ^1^ Department of Nutritional Sciences University of Wisconsin Madison Wisconsin USA; ^2^ School of Biomedical Engineering and Imaging Sciences, King's College London St Thomas' Hospital London UK; ^3^ Department of Radiology University of Wisconsin School of Medicine and Public Health Madison Wisconsin USA

**Keywords:** body composition, computed tomography (CT), mortality prediction, muscle density, muscle quality, myosteatosis, opportunistic imaging

## Abstract

**Background:**

Abdominal CT‐based assessments of skeletal muscle may provide important prognostic information for all‐cause mortality in aging adults. We aimed to evaluate whether AI‐segmented muscle area and muscle density predict long‐term survival in a large, retrospective adult population.

**Methods:**

This retrospective study included 151 141 adult patients who underwent an abdominal CT examination for any indication between 2000 and 2021. A validated automated AI‐based algorithm measured L3‐level muscle area (cm^2^) and muscle density in Hounsfield units (HU). Demographic and clinical data (age, sex, BMI and date of death) were extracted from electronic health records. Survival analyses included Kaplan–Meier curves and multivariable hazard ratio (HR) models to determine the relationship between these muscle metrics and mortality.

**Results:**

Among the 138 535 adults (66 468 men and 72 067 women) included, 28 489 died over the 20‐year period post‐CT, yielding an overall 20‐year survival rate of 0.620 (95% CI: 0.611–0.629). Of these, 9343 deaths occurred within the first year [survival rate: 0.933 (95% CI: 0.932–0.934)]. Mean and median follow‐up time was 6.4 and 4.9 years, respectively. Lower muscle density significantly predicted higher mortality when each patient's measurement was expressed by its percentile within the age and sex‐matched population (HR up to 3.5 in women and 4.0 in men), with decreasing mortality throughout the higher percentiles. Lower muscle area showed a more modest effect on mortality in both sexes when expressed in percentiles. Individuals with high muscle density demonstrated the most favourable survival and those with low density demonstrated the worst survival. Low muscle density significantly predicted mortality across all age groups and both sexes. Conversely, muscle area predicted mortality in all age groups in men, albeit to a lesser degree and did not predict mortality in any age group among women.

**Conclusions:**

Automated CT‐based measurements of muscle density are superior to muscle area in predicting all‐cause mortality in a large, heterogeneous adult population. Incorporating AI‐driven muscle density assessments into routine clinical practice could substantially improve patient risk stratification and management, of particular relevance for aging and sarcopenic patients.

## 1 Introduction

The age‐related loss of muscle mass and function, termed sarcopenia, is increasingly common in older adults. This debilitating muscle health condition leads to profound personal and societal consequences and is only expected to increase within an aging society. As such, sarcopenia presents as a challenging public health concern [1]. Advances in deep learning have facilitated the development of computed tomography (CT) based artificial intelligence (AI) tools that can efficiently derive muscle measures from routine abdominal examinations. The deployment of such advanced methods in very large patient populations will help advance detection and management of various muscle wasting syndromes, including sarcopenia, and define the importance of muscle measures beyond mass alone [2].

Age‐related decline in strength and power precede and occur more rapidly than loss of muscle mass [3, 4, 5]. This disproportionate decline in muscle function is a key determinant of independence and a more consistent predictor of disability and mortality than muscle mass alone in older adults [3, 6]. For this reason, contemporary definitions of sarcopenia have shifted to prioritize muscle quality and function rather than muscle mass alone [7]; muscle quality is defined as the strength per unit muscle mass. However, acquiring volitional measures of muscle function presents challenges in many older adults and necessitates the acquisition of objective imaging biomarkers that associate with muscle function.

CT measured muscle density (i.e., quality) has gained interest as a surrogate for muscle function [8] and has proven to predict health outcomes beyond muscle area [9, 10]. The emergence of automated machine learning CT tools allows for leveraging opportunistic data from screening CT exams to better characterize muscle density in older adults. Previously unachievable, AI CT tools facilitate the analysis of large screening datasets to better define the importance of muscle density as it pertains to survival compared with muscle area in aging adults [2]. The purpose of this study was to evaluate and compare automated AI‐derived CT measures of muscle density and muscle area as predictors of all‐cause mortality in a very large, heterogeneous adult population, stratifying by age and sex.

## 2 Methods

### 2.1 Patient Cohort

**FIGURE 1:**
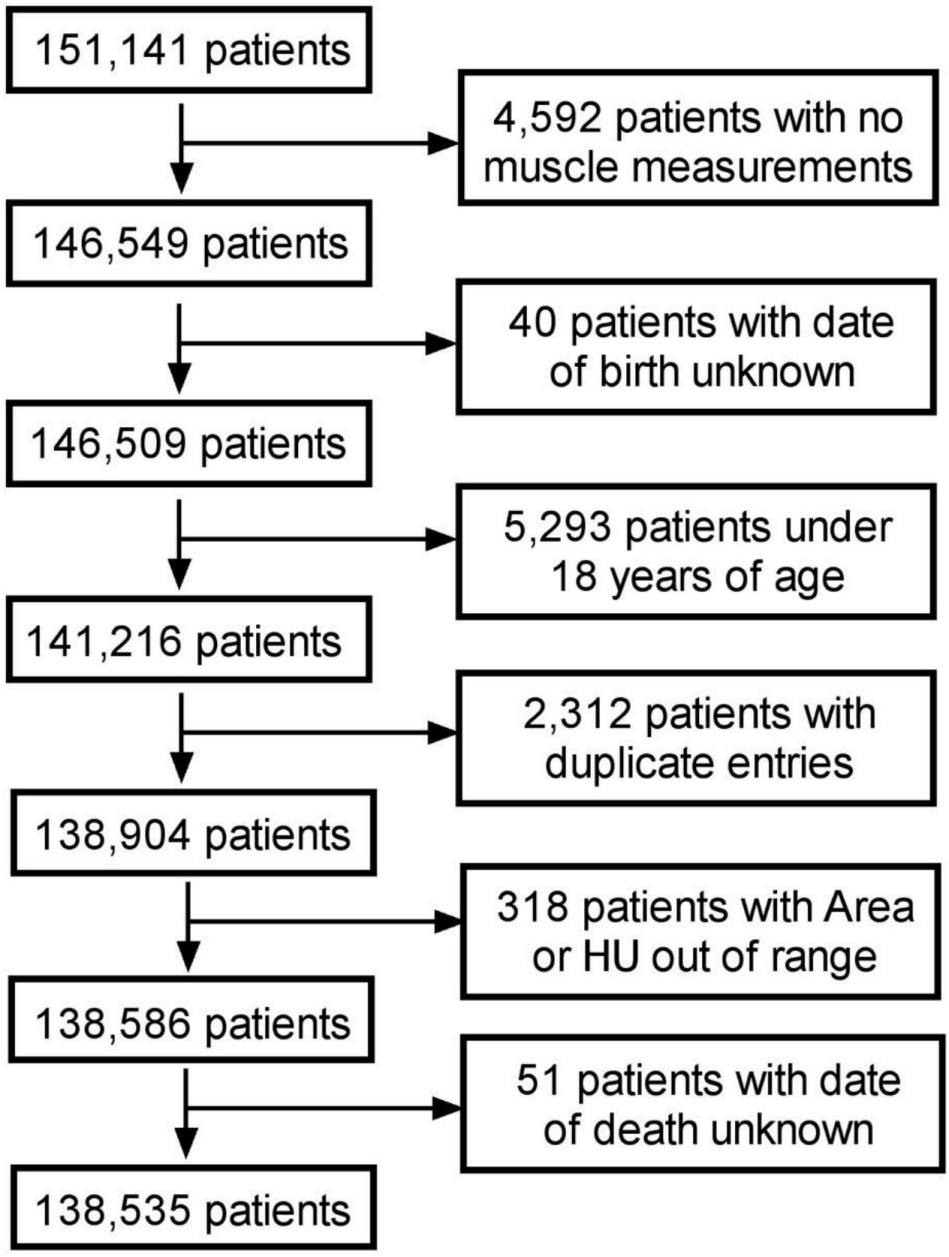
Flowchart explaining the factors for excluding subjects from the final study dataset.

This retrospective study was approved by the Institutional Review Board at the University of Wisconsin and was compliant with the Health Insurance Portability and Accountability Act (HIPAA). The requirement for informed consent was waived due to the retrospective nature of the study. Automated AI‐based algorithms were used to quantify muscle area and muscle density at the third lumbar vertebral (L3) level from abdominal CT scans of 151 141 patients who underwent CT examinations between January 2000 and December 2021. Patients younger than 18 years of age, those with AI algorithm failure, missing or extreme outlier values, or errors in patient death reporting were excluded, resulting in a final study cohort of 138 535 adults (Figure 1). Additional details on the methods used in this automated quantification process have been elaborated on elsewhere [11].

### 2.2 CT Technique

Patients underwent various abdominal CT scanning protocols, which included differing contrast phases, X‐ray tube potentials and dose ranges, utilizing scanners from multiple manufacturers.

### 2.3 Fully Automated AI CT Muscle Biomarkers

AI CT body composition tools have been trained on approximately 44 735 cases across two institutions (UW‐Madison School of Medicine and Public Health and the NIH) and externally validated by numerous prior works [2, 10, 12, 13, 14, 15, 16, 17, 18, 19, 20, 21, 22], including ones external to the training environment [2, 19]. In addition, the technical adequacy of the tool has been validated [23].

The third lumbar vertebral body (L3) level was automatically localized using a convolutional neural network (CNN)‐based unsupervised body part regression (UBR) algorithm. Following localization, muscle tissue at the L3 level was segmented automatically, classifying voxels into muscle or non‐muscle categories based on tissue density and anatomical localization. From the segmented images, muscle area (in cm^2^) and muscle density (in HU) were calculated. Additional details have been previously described [11].

In our full dataset of 151 141 patients, 62.7% of scans were contrast‐enhanced, and 37.3% were non‐contrast. Contrast correction was applied to all contrast‐enhanced scans as follows: muscle area values were adjusted by a factor of 0.9993 (independent of area), and muscle HU values were corrected by a factor of 0.993, linearly. The 0.03% of scans with unknown contrast status were not corrected for enhancement [24].

### 2.4 Clinical Outcomes Measures

Clinical variables including patient age, sex and body mass index (BMI) were extracted from the electronic health record (EHR). The primary clinical outcome was all‐cause mortality. Patient death dates were identified through the EHR. For patients without a recorded death event, the last date of clinical follow‐up recorded in the EHR served as the endpoint.

Given the size of our retrospective dataset and the lack of standardized clinical data across all contributing institutions and time periods, we were unable to reliably extract or validate detailed information on comorbidities, disease acuity, socioeconomic status or lifestyle factors. Similarly, we could not distinguish between patients who were generally healthy and those with chronic or acute disease at the time of imaging.

### 2.5 Statistical Analysis

Analyses were stratified by sex and further divided into four age groups (18–39, 40–59, 60–79 and 80+ years). Kaplan–Meier (KM) survival curves were generated to assess survival probabilities over the 20‐year study period. Patients were grouped by muscle area and muscle density, stratified into high and low categories based on median values for each age group and sex. Hazard ratios (HRs) and corresponding 95% confidence intervals (CIs) were calculated using log‐rank tests to compare survival differences between groups.

Additionally, HRs were examined across percentile groups for muscle area and density to understand the relative risk associated with these metrics over the entire range of data. Statistical analyses were performed using established software (Statistics Kingdom, Melbourne, Australia), and statistical significance was defined as a *p*‐value < 0.05.

## 3 Results

### 3.1 Patient Sample

The final study sample consisted of 138 535 adults (mean age, 53 years; 52% female) who underwent abdominal CT imaging for any reason, as outlined in Figure 1. A total of 28 489 patients died over the 20 years following the CT exam, yielding an overall 20‐year survival rate of 0.620 (95% CI: 0.611–0.629). Of the total deaths, 9343 occurred during the first year (survival rate: 0.933 [0.932–0.934]) and 19 235 occurred within the first 5 years (survival rate: 0.838 [0.835–0.841]). At the 20‐year point, the mean follow‐up time was 6.4 years, and the median follow‐up time was 4.9 years.

At 20 years, survival rates by age group were as follows: 0.917 for ages 18–39, 0.746 for ages 40–59, 0.406 for ages 60–79 and 0.089 for those aged 80 and above. Survival rates were also stratified by sex. Among females, 20‐year survival rates were 0.934 (ages 18–39), 0.783 (40–59), 0.456 (60–79) and 0.101 (80+). Among males, corresponding survival rates were 0.893, 0.703, 0.351 and 0.079, respectively.

### 3.2 Muscle‐Based Body Composition Measures

Patient characteristics by age and body composition measures are summarized in Table 1. The largest age group was 40–59 years (*n* = 52 796). In general, mean muscle HU decreased with age in both men and women. Mean muscle area increased up to age group 40–59 years, at which point it started to decrease for both men and women through age group 80 years plus (Figure S1). BMI followed a similar age‐related pattern as muscle area, increasing through midlife and decreasing in the oldest age group, as shown in Table 1.

**TABLE 1:** Patient characteristics.

Age group (years)	Men					Women				
	*N*	Mean age (SD)	Mean BMI (SD)^a^	Mean muscle area (SD) (cm^2^)	Mean muscle HU (SD)	*N*	Mean age (SD)	Mean BMI (SD)^a^	Mean muscle area (SD) (cm^2^)	Mean muscle HU (SD)
All ages	66 468	53.4 (17.4)	29.0 (6.4)	180.9 (36.8)	33.0 (16.2)	72 067	52.3 (17.7)	29.3 (8.1)	129.3 (28.4)	28.0 (17.9)
Age 18–39	15 685	29.1 (6.3)	27.7 (6.7)	179.4 (36.6)	45.7 (11.0)	18 444	29.1 (6.3)	28.5 (8.4)	129.4 (27.1)	41.5 (12.2)
Age 40–59	24 924	50.6 (5.6)	29.9 (6.8)	188.0 (37.1)	35.3 (13.1)	27 872	50.2 (5.6)	30.0 (8.4)	133.2 (29.0)	30.5 (14.4)
Age 60–79	21 947	68.0 (5.5)	29.3 (6.0)	178.0 (35.3)	24.7 (14.8)	21 112	68.1 (5.6)	29.5 (7.9)	126.7 (28.2)	17.7 (16.3)
Age 80+	3912	84.4 (3.8)	27.3 (4.8)	158.2 (31.0)	14.5 (14.7)	4639	85.3 (4.2)	26.6 (5.8)	116.5 (24.9)	7.2 (15.5)

^a^ 72% of patients with muscle area and HU measurements had a BMI measurement within 365 days of the date of their CT scan.

**FIGURE 2:**
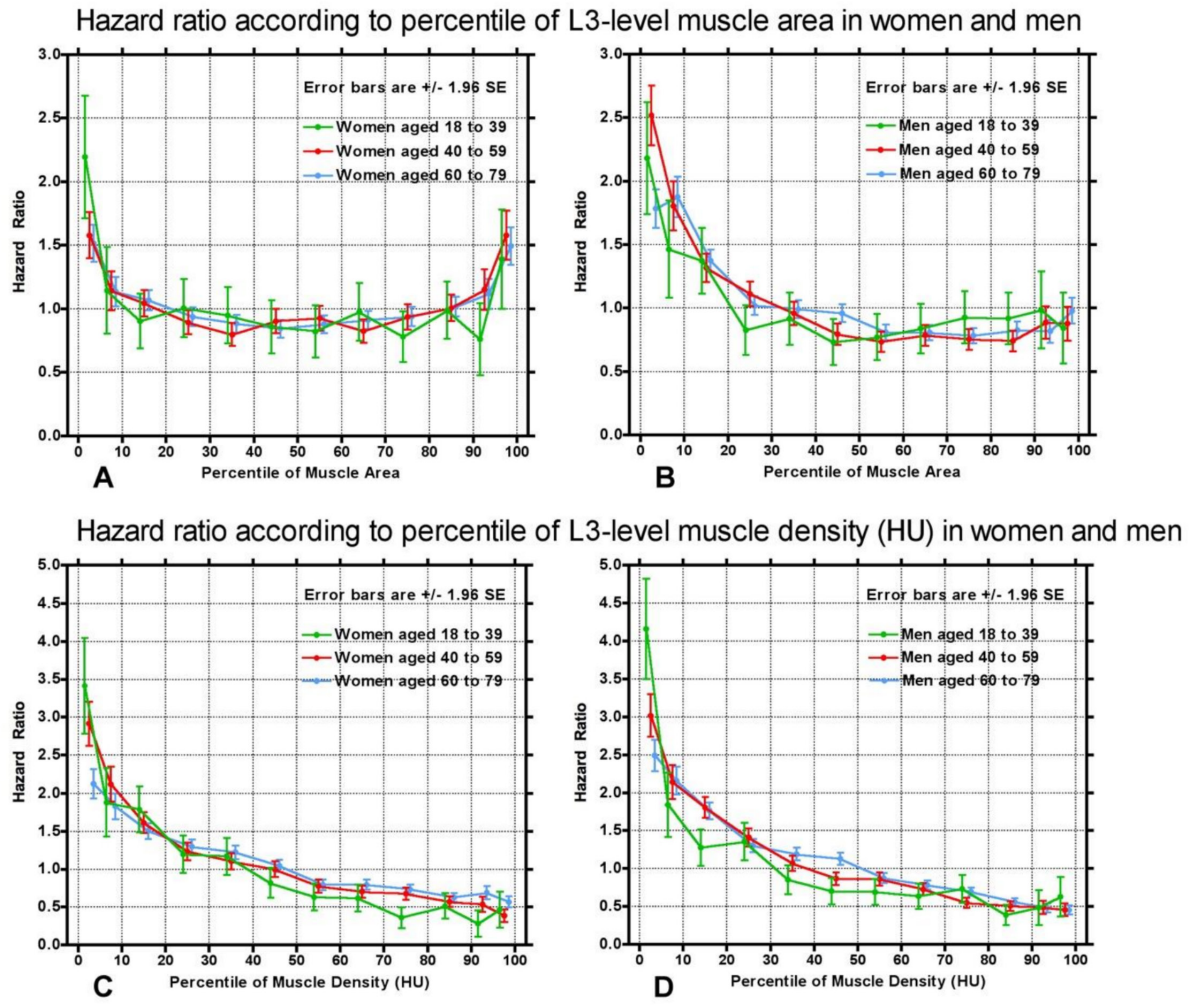
Hazard ratio according to percentile of L3‐muscle area and density in women and men. (A) Relative risk of death in women in three age groups (18–39, 40–59 and 60–79 years) over a 20‐year period as a function of the percentile of the L3‐level muscle area. Points are plotted for the following percentile groups: 0%–5%, 5%–10%, 10%–20%, … … 80%–90%, 90%–95% and 95%–100%. Error bars are ±1.96 SE. (B) Similar plots for men in the same three age groups. (C) Similar plots for women but showing the hazard ratio as a function of the percentile of the L3‐level muscle density (HU). (D) Similar plots for men showing the hazard ratio as a function of the percentile of the L3‐level muscle density (HU).

Relative risk of death post‐CT as a function of muscle area and muscle density are shown in Figure 2. Muscle area in women shows a U‐shaped curve with percentiles below the 10th and above 90th having increased risks of death compared with middle percentiles for all age groups. In men, risk of death follows an L‐shaped curve, with muscle area percentiles below the 20th conferring a higher risk of death for all age groups; the HR of muscle area percentiles > the 20th percentile appear very similar. When considering muscle density, both men and women show a decreasing trend with increasing muscle density. At their peak, the HR approached 2.5 for muscle area and 4.0 for muscle density. Muscle density > the 50th percentile showed a protective effect for both men and women. Muscle area shows a slightly protective effect between the 20th and 90th percentiles for women and between the 20th and 100th percentiles for men.

**FIGURE 3:**
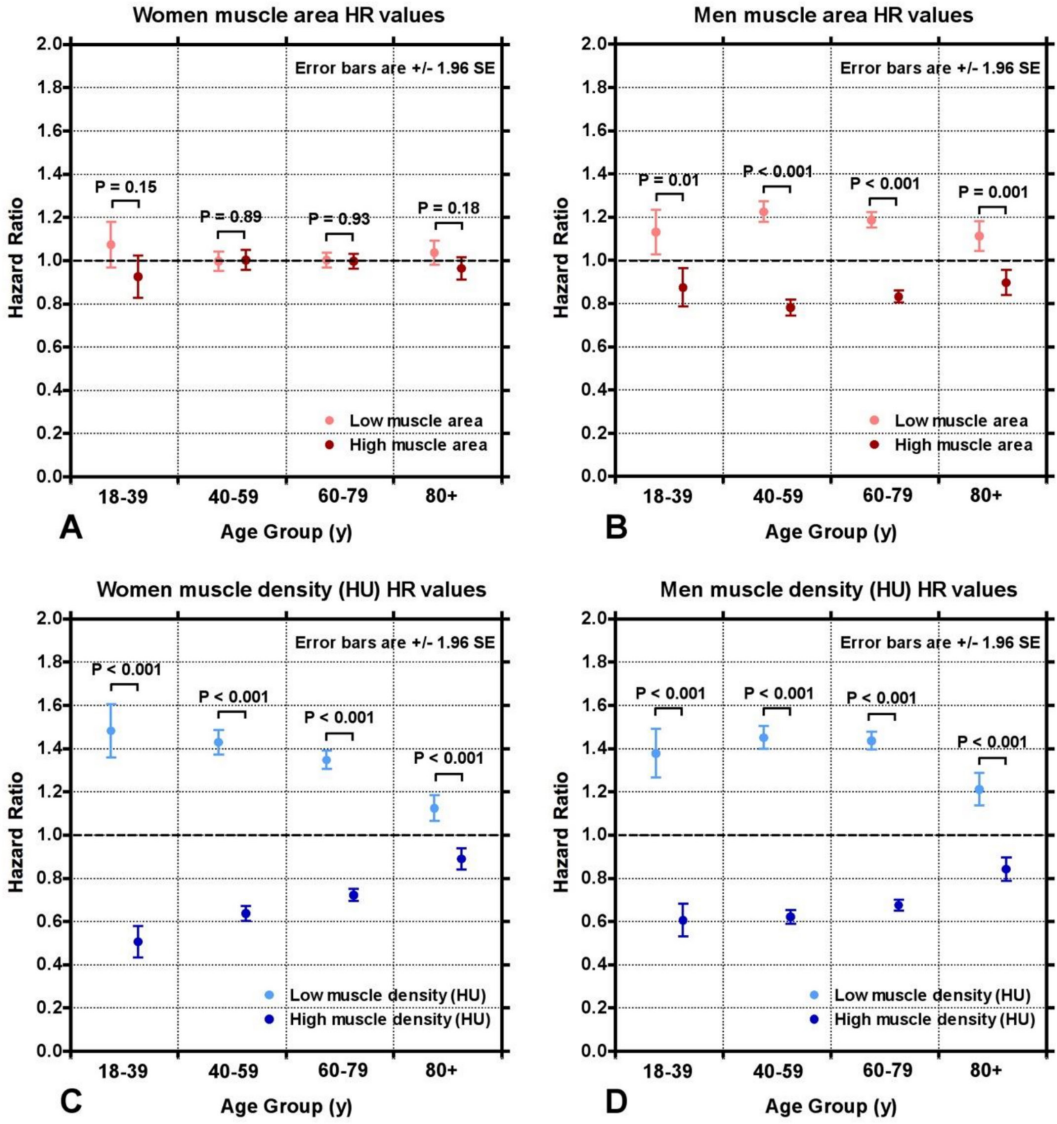
(A) Relative risk of death in women in four age groups (18–39, 40–59, 60–79 and 80+ years) over a 20‐year period for L3‐level muscle areas above and below the median area for their age group. Hazard ratios were calculated separately for each of the four age groups. Error bars are ±1.96 SE. (B) Similar plot for men in the same four age groups. (C) Similar plot for women but showing the hazard ratios for women with L3‐level muscle density (HU) above and below the median for their respective age group. (D) Similar plot to (C) for men.

Figure 3 shows the relative risk of death HR for individuals with high and low muscle area and high and low muscle density. The highest risk of death was in those with low muscle density among all age groups, across men and women. Conversely, high muscle density was protective against risk of death. Low muscle area in men also showed a moderate increased risk of death, albeit to a lesser degree than low muscle density; this effect was not seen in women, with no significant differences in risk of death based on muscle area. The highest risk by age group for women was low muscle density in age group 18–39 years (HR = 1.48, *p* < 0.001), followed by low muscle density in years 40–59, 60–79 and over 80 years (HR = 1.43, 1.35, 1.13, respectively; *p* < 0.001) (Table 2). Men showed similar risks for age groups 18–39 years (HR = 1.38, *p* < 0.001), 40–59 years (HR = 1.45, *p* < 0.001) and 60–79 years (HR = 1.44, *p* < 0.001), with ages 80+ showing a lower, but still increased, risk of death (HR = 1.21, *p* < 0.001). Low muscle area in men was also significant but conferred a smaller risk of death across ages. Muscle area did not confer a significantly different risk of death in women (Table 2).

**TABLE 2:** Hazard ratios and 95% confidence intervals for the different L3 muscle groups.

Age group (years)	Muscle group	Women		Men	
		Hazard ratio (95% CI)	*p*	Hazard ratio (95% CI)	*p*
Age 18–39	Low area	1.073 (0.105)	*p* = 0.17	1.131 (0.103)	***p* = 0.013**
	High area	0.926 (0.098)	*p* = 0.14	0.874 (0.089)	***p* = 0.005**
	Low density (HU)	1.483 (0.123)	***p* < 0.001**	1.379 (0.112)	***p* < 0.001**
	High density (HU)	0.507 (0.073)	***p* < 0.001**	0.607 (0.075)	***p* < 0.001**
Age 40–59	Low area	0.997 (0.045)	*p* = 0.89	1.225 (0.047)	***p* < 0.001**
	High area	1.003 (0.046)	*p* = 0.89	0.781 (0.037)	***p* < 0.001**
	Low density (HU)	1.430 (0.057)	***p* < 0.001**	1.451 (0.053)	***p* < 0.001**
	High density (HU)	0.638 (0.035)	***p* < 0.001**	0.621 (0.032)	***p* < 0.001**
Age 60–79	Low area	1.002 (0.034)	*p* = 0.93	1.187 (0.036)	***p* < 0.001**
	High area	0.998 (0.035)	*p* = 0.93	0.832 (0.028)	***p* < 0.001**
	Low density (HU)	1.348 (0.042)	***p* < 0.001**	1.436 (0.041)	***p* < 0.001**
	High density (HU)	0.73 (0.028)	***p* < 0.001**	0.676 (0.025)	***p* < 0.001**
Age 80–102	Low area	1.037 (0.055)	*p* = 0.18	1.112 (0.068)	***p* = 0.001**
	High area	0.964 (0.052)	*p* = 0.18	0.897 (0.058)	***p* < 0.001**
	Low density (HU)	1.125 (0.059)	***p* < 0.001**	1.212 (0.075)	***p* < 0.001**
	High density (HU)	0.890 (0.049)	***p* < 0.001**	0.843 (0.054)	***p* < 0.001**

*Note:* Bolded *p*‐values represent significance of predicting an increased or decreased risk of death, *p* < 0.05.

**FIGURE 4:**
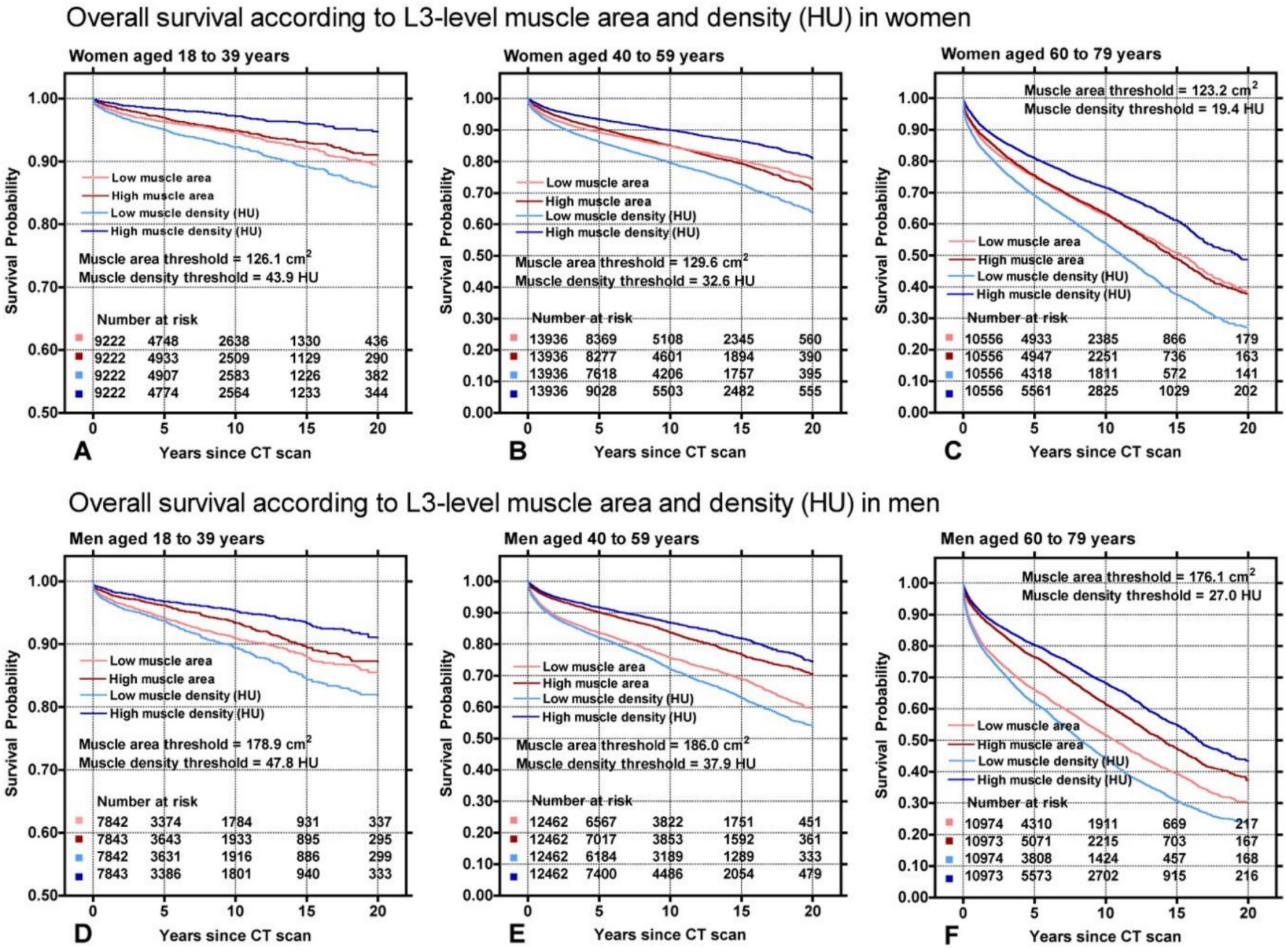
Overall survival according to L3‐level muscle area and density in men and women. Detailed hazard ratios for all groups can be found in Table 2. (A) Kaplan–Meier (KM) survival curves from 0 to 20 years for women aged 18–39 years according to their L3‐level muscle area and density (HU). Low muscle area: women with muscle area below the median for this age group of 126.1 cm^2^; high muscle area: women with muscle area above the median of 126.1 cm^2^; low muscle density: women with muscle density below the median of 43.9 HU; high muscle density: women with muscle density above the median of 43.9 HU. (B) Similar KM survival curves for women aged 40–59 years. For the women in this age group, the muscle area threshold was 129.6 cm^2^ and the muscle density threshold was 32.6 HU. (C) KM curves for women aged 60–79 years. The muscle area threshold was 123.2 cm^2^ and the muscle density threshold was 19.4 HU. (D) KM curves for men aged 18–39 years. For the men in this age group, the muscle area threshold was 178.9 cm^2^ and the muscle density threshold was 47.8 HU. (E) KM curves for men aged 40–59 years. The muscle area threshold was 186.0 cm^2^ and the muscle density threshold was 37.9 HU. (F) KM curves for men aged 60–79 years. The muscle area threshold was 176.1 cm^2^ and the muscle density threshold was 27.0 HU.

KM survival probability curves for ages 18–79 are shown in Figure 4. In all groups, the worst survival is seen in patients with low muscle density (light blue curve), and the best survival is seen in patients with high muscle density (dark blue curve). Higher muscle area (dark red curve) in men shows higher survival than those with low muscle area (light red curve), but that differentiating survival effect is not seen in women. Overall, muscle density had an increased survival discrimination when compared with muscle area in all age groups, in men and women.

## 4 Discussion

In this study, an AI CT tool was used to measure muscle area and density in 138 535 adults having an abdominal CT exam. When inspecting relative risk of death over a 20‐year time period, muscle density shows a decreasing HR trend with increasing muscle density throughout the entire percentile range of 0–100th. This trend persisted between sexes and in all age groups. In general, muscle area conferred a much lower risk of death throughout the range of percentiles. When separating by high or low muscle density and area, the highest risk of death was in those with low muscle density and those with the highest muscle density had the lowest risk of death. Although a modest trend to a higher risk of death with low muscle area was shown in men, high or low muscle area had little or no effect on the relative risk in women. Twenty‐year survival curves consistently showed that high muscle density was associated with the highest survival rates, while low muscle density was associated with the lowest survival rates; muscle area failed to show adequate survival discrimination in women and showed very little discrimination in men. These findings highlight the importance of muscle density in predicting survival and the need to incorporate muscle density in the risk management of older adults.

The loss of muscle area or mass has long been considered one of the most consequential changes associated with aging. Although low muscle mass, and those diagnosed with sarcopenia, are at risk for a number of consequential outcomes, multiple large studies indicate that low muscle density is a more potent predictor of reduced function and survival [9, 25]. In an asymptomatic outpatient adult cohort (~9000 patients undergoing CT colonography screening), Pickhardt et al. [9] found low muscle density conferred a far higher risk of 10‐year mortality (unadjusted HR ~4.3) than low muscle cross‐sectional area (HR ~1.8). After adjusting for covariates, only muscle density remained an independent predictor (adjusted HR ~1.9). Similarly, in a Medicare patient sample, each standard deviation (SD) decrease in total muscle attenuation was associated with a ~46% increase in 1‐year mortality risk, compared with ~31% increased risk per SD decrease in muscle area [26]. These findings align with a multi‐ethnic community cohort over a 10‐year follow‐up where greater abdominal muscle density was strongly protective. Men in the highest quartile of density had a 73% reduction in mortality risk compared with those in the lowest quartile [27]. By contrast, muscle area showed little or no protective effect. Notably, these associations held true across age groups, suggesting that muscle density's prognostic value is robust from younger to older adults. These data align with a recent publication showing muscle density as the major determinant for a CT‐derived biological aging model [12]. Together, these findings suggest that muscle mass may not uniformly convey mortality risk across populations. In our cohort, low muscle mass in women showed little survival impact.

This dimorphism in our results may reflect sex‐specific biological mechanisms that differentially affect age‐related muscle remodelling. Experimental and human studies indicate that sex steroids modulate muscle protein turnover, mitochondrial function and fat partitioning in a sex‐dependent manner [28, 29, 30, 31], which could translate into greater susceptibility to increased myosteatosis and loss of muscle quality in some individuals despite relatively preserved muscle mass. In addition, men and women differ in muscle fibre‐type distribution and contractile gene expression trajectories with aging, with males showing more pronounced alterations in oxidative phosphorylation pathways and females demonstrating distinct AKT‐mediated growth signalling, suggesting that similar aging processes may operate with different magnitudes and consequences across sexes [32, 33]. CT‐derived muscle density is strongly influenced by inter‐ and intramuscular adipose tissue deposition, and prior work has reported sex‐specific CT muscle and fat phenotypes linked to clinical outcomes in various populations [3, 34, 35]. These observations support the hypothesis that sex‐related differences in hormonal milieu, fibre composition and intramuscular fat accumulation could partly explain why muscle density, rather than muscle area, consistently predicts mortality across both sexes in our cohort, and they underscore the need for future mechanistic and longitudinal imaging studies specifically designed to disentangle sexual dimorphism in muscle quantity versus quality across the lifespan.

The protective impact of high muscle quality is also evident in multiple clinical populations. In oncologic and critical‐care populations, patients with myosteatosis (i.e., increased muscle fat infiltration) have worse prognosis than those without myosteatosis [36]. In older adults admitted for a hip fracture, each SD increase in paraspinal muscle density conferred ~11%–18% lower 5‐year mortality risk, independent of muscle size and comorbidities [37]. Retaining muscle density and avoiding myosteatosis, seems to impart a longevity benefit, as a buffer against mortality even in the setting of low muscle area [27]. This protective effect likely stems from better metabolic health, strength and function in individuals with low fat infiltration in their muscle. While there is a small amount of contradictory research [38], the majority of accumulating evidence from CT‐based analyses suggest muscle density, as a surrogate of quality, outperforms muscle quantity as a predictor of poor outcomes.

The use of AI‐driven tools for quantifying muscle density from CT imaging presents an opportunity to uncover novel prognostic markers of health and disease. These methods enable rapid, reproducible and scalable assessment of muscle quality, with potential applications in personalized medicine and population‐level risk stratification. However, several limitations of this study should be considered. First, this was a retrospective analysis of individuals who underwent abdominal CT imaging for a wide range of clinical indications. As such, the cohort includes patients with both acute and chronic conditions, as well as those undergoing routine screening, introducing heterogeneity in baseline health status. While this diversity enhances generalizability, it may also introduce variability in muscle density that is not solely attributable to aging or chronic disease. Inter‐ and intramuscular fat accumulation, however, occurs across both diseased and healthy aging populations and has been confirmed through histology [39, 40, 41, 42]. CT‐based attenuation measures show a strong correlation with this infiltration, yet the specific drivers—whether metabolic, inflammatory and/or related to physical inactivity—remain to be fully elucidated [43]. Although reduced muscle density, reflected by lower HU, is strongly associated with fat infiltration into skeletal muscle, the biological mechanisms underlying this process are not fully understood [44]. Furthermore, the dataset comprises both contrast‐enhanced and non‐contrast CT scans. Although prior work has validated the use of correction algorithms to harmonize muscle density measurements across contrast types, subtle differences may persist due to contrast perfusion effects [24]. Further investigation is warranted to quantify the impact of contrast phase, if any, on muscle attenuation values. Additionally, the scale of this retrospective dataset precluded the collection of detailed clinical variables, including comorbidities, malnutrition, lifestyle, and their impact on muscle density. Although BMI data were available for 72% of the cohort, it was not included in multivariable models. BMI was not measured concurrently with the CT examination and was instead captured within a range of ±365 days, which may introduce misclassification and bias. This limitation is particularly relevant in older adults, where weight and body composition can change rapidly in the setting of illness or functional decline. We decided to focus our data on the simplicity and direct clinical applicability of raw CT values as independent imaging biomarkers, without requiring additional clinical variables. Lastly, the demographic composition of our cohort was predominantly white, limiting the ability to evaluate potential differences in muscle density and its clinical relevance across racial and ethnic groups. Future studies should prioritize more diverse, multicentre populations that reflect broader racial and ethnic representation to assess the generalizability of muscle‐based biomarkers and ensure equitable clinical translation.

In conclusion, automated AI‐based CT body composition tools to derive muscle measures can be utilized to assess risk in large cohorts. Low muscle density is an important predictor of mortality risk regardless of muscle area. These findings highlight the importance of muscle quality attributes that decrease its density, reflecting poor functioning muscle, rather than muscle quantity. Implementation of these tools provides a more comprehensive approach for identifying risk burden in an increasingly older society. Finally, these data provide evidence to support a new approach that defines sarcopenia with CT‐derived age‐ and sex‐based cut‐points of muscle quantity and muscle quality (i.e., density), as sought by the sarcopenia field.

## Funding

The authors have nothing to report. The authors confirm that all resources were provided by their respective institutions.

## Disclosure

Dr. Pickhardt is an advisor to Nanox, Bracco, GE Healthcare and ColoWatch. Dr. Garrett is an advisor to RadUnity and a shareholder in NVIDIA. All other authors have no disclosures.

## Conflicts of Interest

The authors declare no conflicts of interest.

## Supporting information


**Figure S1:** Box‐and‐whisker plots showing the cross‐sectional muscle area and the mean density in Hounsfield Units (HU) in 10‐year age groups at the level of the L3 vertebra. (A) Muscle area for men; (B) muscle area for women; (C) mean muscle HU for men; (D) mean muscle HU for women. Age groups are from 20 to 29 years to age 90+. The box‐and‐whisker plots show the median and interquartile range, with the whiskers marking the 5th and 95th percentiles. The numbers in the area plots indicate the number of subjects in each age bracket.
