# Supplementary material for: Automated CT‐Based Muscle Density Predicts Mortality Regardless of Muscle Area
Source: J Cachexia Sarcopenia Muscle. 2026 Apr 2;17(2):e70268. doi: 10.1002/jcsm.70268 (PMC13045369; doi:10.1002/jcsm.70268)
Supplement: Supplementary file 1 — Figure S1: Box‐and‐whisker plots showing the cross‐sectional muscle area and the mean density in Hounsfield Units (HU) in 10‐year age groups at the level of the L3 vertebra. (A) Muscle area for men; (B) muscle area for women; (C) mean muscle HU for men; (D) mean muscle HU for women. Age groups are from 20 to 29 years to age 90+. The box‐and‐whisker plots show the median and interquartile range, with the whiskers marking the 5th and 95th percentiles. The numbers in the area plots indicate the number of subjects in each age bracket. [file JCSM-17-e70268-s001.docx]

**Automated CT-based muscle density predicts mortality regardless of muscle area**

**In preparation for submission to: Journal of Cachexia, Sarcopenia, and Muscle**

Adam J. Kuchnia, PhD^a^

Glen M. Blake, PhD^b^

Matthew H. Lee, MD^c^

Jevin Lortie, PhD^a^

Rachel Fenske, PhD^a^

John W. Garrett, PhD^c^

Perry J. Pickhardt, MD^c^

^a^ Department of Nutritional Sciences, University of Wisconsin, Madison, Wisconsin, United States of America

^b^ School of Biomedical Engineering and Imaging Sciences, King’s College London, St Thomas’ Hospital, London, United Kingdom

^c^ Department of Radiology, University of Wisconsin School of Medicine and Public Health, Madison, Wisconsin, United States of America

Disclosures: Dr. Pickhardt is an advisor to Nanox, Bracco, GE Healthcare and ColoWatch. Dr. Garrett is an advisor to RadUnity and a shareholder in NVIDIA. All other authors have no disclosures.

Corresponding Author:

Perry J. Pickhardt

[ppickhardt2@uwhealth.org](mailto:ppickhardt2@uwhealth.org)

**Supplemental Figure**

**Figure S1**


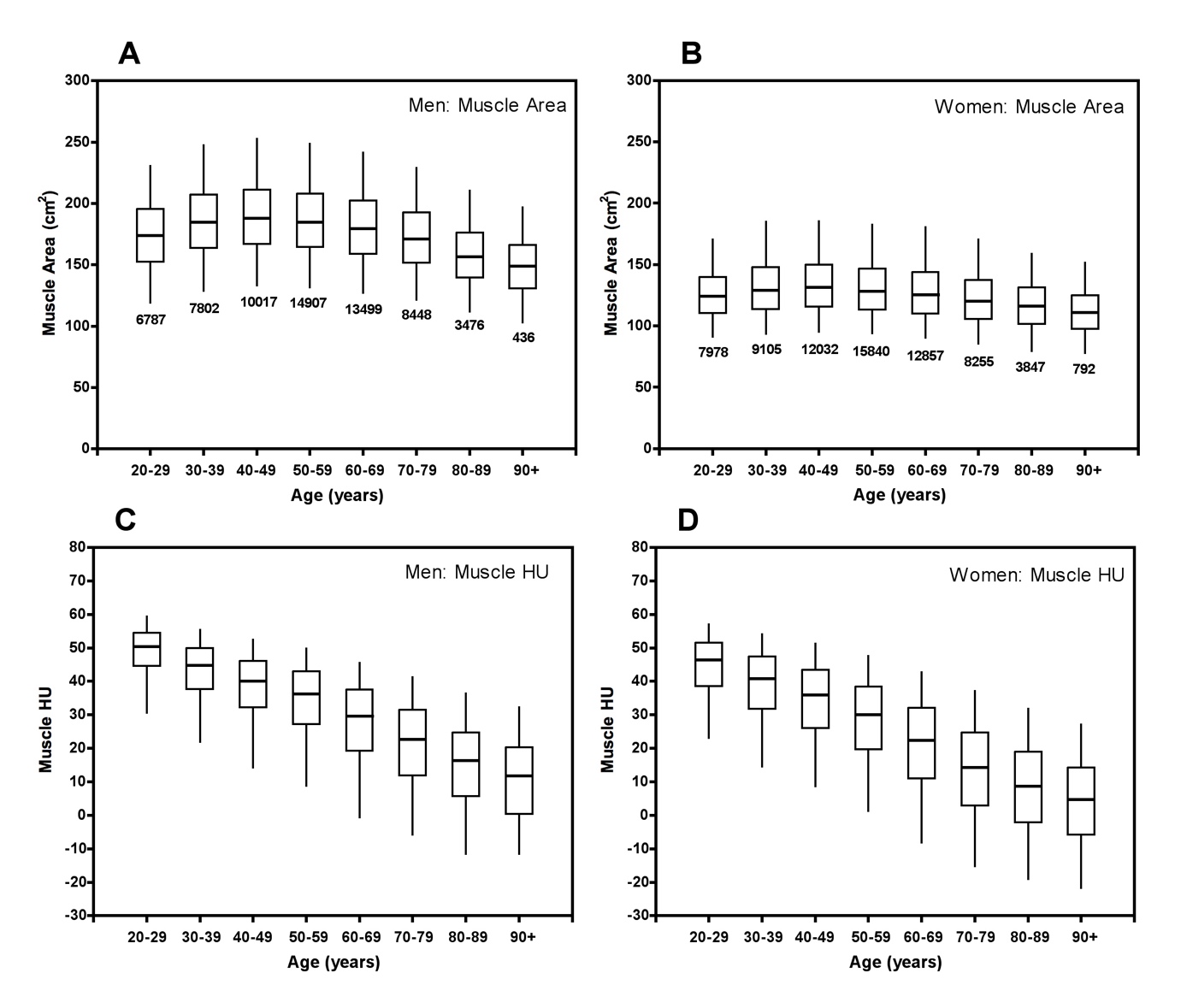


Box-and-whisker plots showing the cross-sectional muscle area and the mean density in Hounsfield Units (HU) in 10-year age groups at the level of the L3 vertebra. (A) Muscle area for men; (B) Muscle area for women; (C) Mean muscle HU for men; (D) Mean muscle HU for women. Age groups are from 20 to 29 years to age 90+. The box-and-whisker plots show the median and interquartile range, with the whiskers marking the 5^th^ and 95^th^ percentiles. The numbers in the area plots indicate the number of subjects in each age bracket.
